# Is upfront stereotactic radiosurgery a rational treatment option for very elderly patients with brain metastases? A retrospective analysis of 106 consecutive patients age 80 years and older

**DOI:** 10.1186/s12885-016-2983-9

**Published:** 2016-12-15

**Authors:** Shoji Yomo, Motohiro Hayashi

**Affiliations:** 1 Division of Radiation Oncology, Aizawa Comprehensive Cancer Center, Aizawa Hospital, 2-5-1, Honjo, Matsumoto-city, Nagano-prefecture 390-0814 Japan; 2 Saitama Gamma Knife Center, San-ai Hospital, Saitama-city, Saitama-prefecture Japan

**Keywords:** Brain metastases, Elderly patients, Stereotactic radiosurgery, Gamma knife

## Abstract

**Background:**

Advanced age has been shown to be a factor predicting poor survival in patients with brain metastases (BM). There have been only a few studies focusing on stereotactic radiosurgery (SRS) for elderly BM patients. The present study aimed to investigate the efficacy and limitations of SRS for very elderly BM patients.

**Methods:**

This was a retrospective observational study analyzing 106 consecutive patients (69 males/37 females) age 80 years and older who received upfront Gamma Knife SRS for BM between January 2009 and October 2015. The median age was 84 years, and the median Karnofsky performance status (KPS) was 70. Fifty-two patients had a solitary BM, and others multiple BM. The median cumulative tumor volume was 3.9 mL and the median dose prescribed was 20 Gy. Overall survival (OS), neurological death rates and distant and local intracranial tumor control rates were analyzed.

**Results:**

No patients were lost to follow-up. Six-month and 12-month OS rates were 54% and 32%, respectively. The median OS time was 7.1 months. Competing risks analysis showed that 6-month and 12-month neurological death rates were 8% and 11%, respectively. In total, 245 / 311 tumors (79%) in 82 patients (77%) with sufficient radiological follow-up data were evaluated. Six-month and 12-month distant BM recurrence rates (per patient) after SRS were 17% and 25%, respectively. Six-month and 12-month rates of local tumor control (per lesion) were 94% and 89%, respectively. Repeat SRS, salvage WBRT and surgical resection were subsequently required in 25, 4 and 1 patient, respectively. Proportional hazard regression analysis showed that KPS ≥ 70 (HR: 0.444, *P* < .001), controlled primary disease/no extracranial metastases (HR: 0.361, *P* < .001) and female sex (HR: 0.569, *P* = 0.028) were independent factors predicting better OS. Similarly, tumor volume (>2 mL) was the only factor predicting a higher rate of local control failure (HR: 12.8, *P* = 0.003).

**Conclusions:**

The present study suggested an upfront SRS strategy to offer a feasible and effective treatment option for very elderly patients with limited BM. In the majority of patients, neurological death could be delayed or even prevented.

## Background

In industrial nations, demographic projections portend a substantial increase in numbers of older persons, thus implying consequent increases in cancer incidence and mortality in the elderly [1]. Advanced age has been shown to be an important prognostic factor for survival in patients with brain metastases (BM) [2–6]. Diminished performance status and the presence of co-morbidities may make radiotherapy less feasible in the elderly. Moreover, elderly patients may prefer less aggressive treatment for BM. In fact, palliative whole brain radiotherapy (WBRT) utilization rates drop steeply in the elderly [7]. Recently, in selected patients, WBRT has been omitted from the initial management for BM with the aim of reducing the potential risk of delayed neurological toxicity [8–10]. Stereotactic radiosurgery (SRS) has emerged as the preferred treatment modality, either alone or in combination with other modalities [10, 11]. The delivery of highly focused radiation with a sharp dose fall-off is theoretically expected to reduce delayed neurotoxicity, and this feature makes it applicable in both the upfront and the salvage setting. To date, a few studies have investigated SRS treatment results for elderly patients with BM, but the definitions of elderly patients differed among these prior SRS studies (Table 1) [12–16]. We consider evidence for the clinical efficacy of SRS for elderly patients with BM to still be insufficient and advocate additional research to confirm the therapeutic benefits of SRS in this population.

**Table 1:** Series of treatment outcomes of elderly patients undergoing SRS for BM

First author & year	Treatment modality	No. of patients	Median age (Cut-off age)	No. receiving prior WBRT (%)	MST after SRS (months)	Factors predicting longer OS	Local tumor control	Remote brain recurrence
Noël, 2005 [12]	LINAC	117	71 years (≥65 years)	18 (15%)	8	KPS ≥ 70	98%/6 months 91%/2 years	33%/6 months 60%/2 years
Kim, 2008 [13]	LINAC/GK	44	79 years (≥75 years)	17 (39%)	^a^7.3	Single BM	^b^88%	^b^81%
Minniti, 2013 [14]	LINAC	102	77 years (≥70 years)	0 (0%)	13.2	KPS > 70, Stable extracranial disease	90%/1 year 84%/2 years	54%/1 year 78%/2 years
Watanabe, 2014 [15]	GK	165	82 years (≥80 years)	4 (2%)	5.3	NR	^b^95%	NR
Park, 2015 [16]	GK	147	79 years (≥70 years)	13 (9%)	5.6	KPS ≥ 90, No extracranial metastasis	NR	NR
Present study, 2016	GK	106	84 years (≥80 years)	0 (0%)	7.1	KPS ≥ 70, Controlled primary/no extracranial metastasis, Female sex	94%/6 months 89%/1 year	17%/6 months 25%/1 year

*SRS* stereotactic radiosurgery, *BM* brain metastasis, *WBRT* whole brain radiotherapy, *MST* median survival time, *LINAC* linear accelerator, *GK* gamma knife, ^a^time from the diagnosis, ^b^crude value, NR not reported

Thus, the efficacy and limitations of our SRS-oriented treatment strategy for very elderly patients, i.e. those at least 80 years of age, with newly diagnosed and/or recurrent BM were investigated. The present study also explored factors predicting the survival of elderly patients undergoing SRS.

## Methods

### Patient population

The present study was conducted in compliance with the Declaration of Helsinki (sixth revision, 2008), and fulfilled all of the requirements for patient anonymity. The Aizawa Hospital Institutional Review Board approved this retrospective clinical study in October 2015 (No. 2015–038).

We analyzed our prospectively maintained institutional radiosurgical database to examine the radiological and clinical outcomes. Between December 2008 and October 2015, 106 consecutive very elderly patients with BM who underwent Gamma Knife SRS as upfront treatment were eligible for the present study. During this study period, 2 patients receiving prior WBRT before SRS were identified and excluded. Of the eligible patients, 69 were male and 37 were female. The median age was 84 years (range: 80–93 years). The median Karnofsky performance status (KPS) at the time of SRS was 70 (range: 30–100). The primary cancers were of the lung in 74 patients (including 8 with small cell lung cancer), the digestive tract in 16, melanoma in 3, the breast in 2, the kidney in 2, the thyroid in 2, the ovary in 1, and were of unknown origin in 6 patients. Before SRS, 7 patients had undergone microsurgical resection of BM and one had received an endoscopic third ventriculostomy for obstructive hydrocephalus. The median interval between primary diagnosis and SRS was 11.3 months (range: 0–246 months). Patient characteristics are summarized in Table 2.

**Table 2:** Summary of clinical data from 106 consecutive patients

Characteristics	Overall (*n* = 106)
Sex (male/female)	69/37
Age (years), median (range)	84 (80–93)
KPS, median (range)	70 (30–100)
Controlled primary disease and no extracranial metastases	25 (24%)
RTOG-RPA class II/class III	56/50
Ongoing systemic chemotherapy	31 (29%)
Time from primary diagnosis to initial SRS (months), median (range)	11.3 (0–246)
Cumulative TV on initial SRS (mL), median (range)	3.9 (0.2–53.3)
No. of intracranial lesions at initial SRS, median (range)	2 (1–16)
No. of patients treated with 2-session SRS	15 (14%)

*KPS* Karnofsky performance status, *RTOG* radiation treatment oncology group, *RPA* recursive partitioning analysis, *SRS* stereotactic radiosurgery, *TV* tumor volume

### Radiosurgical Indications and Techniques

One hundred and three (97%) patients included in the present study had been diagnosed and their primary tumors treated at the referring regional hospitals, whose own cancer boards had provisionally determined the appropriateness of SRS. The patients were then referred to our institution to receive SRS for BM. The remaining three patients had been treated at our institution. The SRS protocol used in this study was based on the standard care established at our institution. Patients with up to ten BM principally received SRS. Providing that WBRT had been refused by the patient, SRS was applied for multiple BM, even in cases with more than 10 lesions, when the patient’s systemic condition was such that SRS intervention would be tolerable and fully informed consent for treatment had been obtained. Surgical resection was, in principle, indicated for large tumors with a mass effect. If surgery did not seem feasible due to a poor prognosis or advanced systemic disease, 2-session SRS was indicated for carefully selected large tumors (≥10 mL) [17].

SRS was performed using the Leksell G stereotactic frame (Elekta Instruments, Stockholm, Sweden). The frame was placed on the patient’s head under local anesthesia supplemented with mild sedation. Three-dimensional volumetric gadolinium-enhanced T1-weighted magnetic resonance (MR) images, 2 mm in thickness, T2-weighted MR images and contrast-enhanced computed tomography covering the whole brain were routinely used to generate a treatment plan with Leksell Gamma Plan software (Elekta Instruments). Prescribed doses were selected in principle according to the dose protocol of the JLGK 0901 study [10]. The technical details of 2-session SRS were previously described in detail [17]. Total prescription doses in 2-session SRS were recalculated into a single dose applying a linear-quadratic (LQ) model by assuming the alpha/beta ratio to be 10 for BM [18]. All treatments were performed with the Leksell Gamma Knife Model C or Perfexion.

### Post-SRS Management and Follow-up Evaluation

Clinical follow-up data as well as contrast-enhanced MR images were obtained every one to three months. If metachronous distant metastases were identified, they were, in principle, managed with repeat SRS. When miliary metastases (numerous tiny enhanced lesions) and/or leptomeningeal carcinomatosis was newly documented, WBRT was then recommended. Local control failure was defined as an at least 20% increase in the diameter of the targeted lesions, taking as a reference the pre-SRS diameter, irrespective of whether the lesion was a true recurrence or delayed radiation injury. We endeavored to meticulously differentiate delayed radiation injury from tumor recurrence, based on serial MR imaging findings [19] and the clinical course. Additional SRS was possible provided that the volume of the local tumor recurrence was small enough for single-dose SRS. Surgical removal was indicated when neurological signs became refractory to conservative management, regardless of whether the radiological diagnosis was local tumor progression or radiation necrosis. Any adverse events attributable to SRS procedures were evaluated based on the National Cancer Institute Common Terminology Criteria for Adverse Events (NCI-CTCAE; ver.4.0).

Before closing the research database for analysis in August 2016, the authors updated the follow-up data of patients who had not visited our outpatient department for more than three months. Inquiries about the date and mode of death were made by directly corresponding with the referring physician and/or the family of the deceased patient, with written permission obtained at the time of undertaking SRS from all patients and/or their relatives, allowing the use of personal data for clinical research. Neurological death was defined as death attributable to central nervous system metastases including tumor recurrence and carcinomatous meningitis. Deaths with unspecified causes were also categorized as neurological deaths in the present study.

### Statistical analysis

The overall survival (OS) rate was calculated by the Kaplan-Meier product limit method. The neurological and non-neurological death rates were calculated employing Gray’s test [20], wherein each event was regarded as a competing risk for another event. For the estimation of local control failure rates and distant BM recurrence, Gray’s test was similarly applied, with subsequent WBRT for distant recurrence and the patient’s death being regarded as competing events, respectively. All of the above analyses were based on the interval from the date of initial SRS treatment until the date of each event. The Cox and Fine-Gray proportional hazards models [21] were appropriately employed to investigate prognostic factors associated with OS and neurological death-free survival, and for local tumor control. Potential prognostic factors were selected with reference to other SRS series [12–14, 16, 22, 23]. The statistical processing software package “R” version 3.0.1 (The R Foundation for Statistical Computing, Vienna, Austria) was used for all statistical analyses. A *P*-value < 0.05 was considered to indicate a statistically significant difference.

## Results

Eighty-one patients (76%) had active systemic disease and/or extracranial metastases and 31 (29%) were receiving systemic chemotherapy around the time of the initial SRS. Sixteen patients (15%) received molecular targeted therapy, and 12 patients with epidermal growth factor mutation positive lung adenocarcinoma were administered oral tyrosine kinase inhibitors. Fifty-two patients (49%) had solitary BM. The median number of BM at the initial SRS was 2 (range: 1–16 tumors). In total, 311 tumors were being treated at the time of the initial SRS. The median tumor volume (TV) was 0.4 mL (range: 0.01–53.3 mL) and the median cumulative TV was 3.9 mL (range: 0.2–53.3 mL). The median prescribed dose for single-session SRS was 20 Gy (range: 15–22 Gy). Fifteen patients (14%) with 17 large tumors were allocated to 2-session SRS. The median TV of large BM treated with 2-session SRS was 17.9 mL (range: 10.1–53.3) and the median cumulative dose prescribed was 27 Gy (range: 20–28). By the time of the second session, the median TV had been reduced to 8.6 mL (range: 2.3–42.6).

**Fig. 1:**
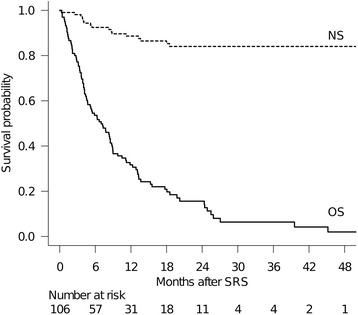
Survival results for very elderly (≥80 years of age) patients with BM treated with SRS. The solid line represents overall survival (OS) probability. The median survival time (MST) was 7.1 months (95% CI: 4.6–8.7). Six-month and 12-month OS rates after SRS were 54% and 31%, respectively. The dotted line represents the neurological death-free survival (NS) probability adjusted for competing events. Six-month and 12-month NS rates after SRS were 92% and 89%, respectively. Note that the distance between these two lines, NS and OS, represents the cumulative incidence of non-neurological death

**Fig. 2:**
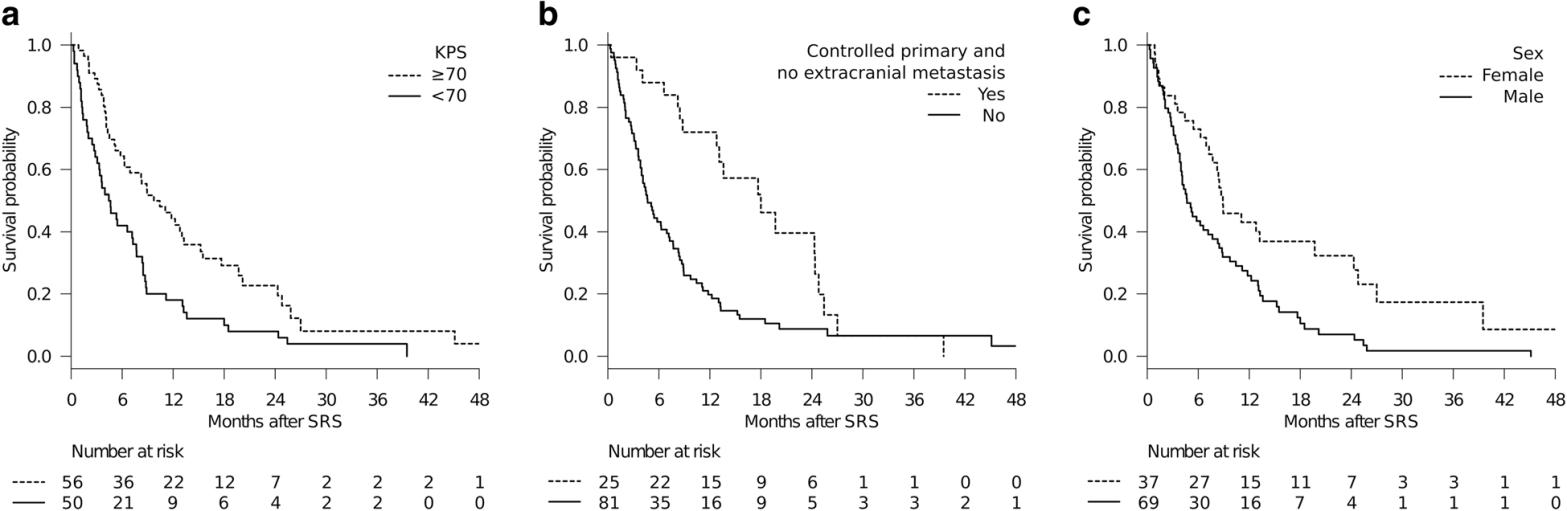
Overall survival (OS) results stratified according to independent prognostic factors. KPS ≥ 70 (Hazard ratio (HR): 0.444, *P* < .001) (**a**). Controlled primary disease/no extracranial metastases (HR: 0.361, *P* < .001) (**b**). Female sex (HR: 0.569, *P* = 0.028) (**c**)

Full clinical results were available for all 106 patients as follow-up data had been completely updated in all patients. The median follow-up time after SRS was 7.1 months (range: 0.3–64 months). At the time of assessment, 11 patients (10%) were alive and 95 (90%) had died. The cause of death in 77 patients was progressive systemic cancer or related complications (e.g.: acute respiratory failure, liver insufficiency) and one patient died from severe head trauma not associated with either the systemic cancer or BM. Eleven patients died as a consequence of their BM and in the remaining 6 the cause of death could not be specified. The median survival time (MST) was 7.1 months (95% CI: 4.6–8.7). Six-month and 12-month OS rates after SRS were 54% and 32%, respectively (Fig. 1). Eleven of these 106 (10%) elderly patients survived more than 2 years after SRS. The Cox proportional hazards model for OS identified KPS ≥ 70 (Hazard ratio (HR): 0.444, 95% confidence interval (CI): 0.284–0.715, *P* < .001) (Fig. 2a), controlled primary and no extracranial metastasis (HR: 0.361, 95% CI: 0.206–0.632, *P* < .001) (Fig. 2b) and female sex (HR: 0.569, 95% CI: 0.344–0.942, *P* = 0.028) (Fig. 2c) as favorable factors independently predicting better OS rates (Table 3). Six-month and 12-month neurological death probabilities adjusted for competing events (non-neurological death) were 8% and 11%, respectively (Fig. 1). No factors were identified as being statistically significantly associated with higher risk of neurological death by the Fine-Gray proportional hazards model (Table 4).

**Table 3:** Analysis of factors predicting patient survival after SRS (Cox proportional hazards model)

Covariate (No. of patients)	MST (months)	*P* value	Hazard ratio (95% CI)
Sex		0.028	0.569 (0.344–0.942)
Female (37)	8.9		
Male (69)	4.7		
KPS		< .001	0.444 (0.279–0.706)
KPS ≥ 70 (56)	9.7		
KPS < 70 (50)	4.5		
Controlled systemic disease/no extracranial metastasis		< .001	0.361 (0.206–0.632)
Yes (25)	18.0		
No (81)	4.7		
Interval from primary diagnosis to SRS		0.053	0.687 (0.448–1.05)
Long > 12 months (54)	8.8		
Short ≤ 12 months (52)	4.4		
Chemotherapy		0.806	1.08 (0.603–1.92)
Yes (31)	12.8		
No (75)	5.2		
No. of metastases		0.513	0.864 (0.557–1.34)
Solitary (52)	6.9		
Multiple (54)	7.1		
Cumulative PIV		0.375	1.25 (0.762–2.06)
Small ≤ 5 mL (45)	7.7		
Large > 5 mL (61)	5.4		
Type of intervention		0.269	1.47 (0.744–2.89)
Two-session (15)	4.0		
Single session (91)	7.2		

*SRS* stereotactic radiosurgery, *MST* median survival time, *CI* confidence interval, *KPS* Karnofsky performance scale, *PIV* prescription isodose volume

**Table 4:** Analysis of factors predicting neurological death-free survival after SRS (Fine-Gray proportional hazards model)

Covariate	*P* value	Hazard ratio (95% CI)
Female Sex	0.20	0.443 (0.129–1.53)
High KPS (≥70)	0.43	0.656 (0.231–1.86)
Controlled systemic disease/no extracranial metastasis	0.62	1.35 (0.417–4.35)
Long interval from primary diagnosis to SRS (>12 months)	0.48	1.41 (0.549–3.60)
Chemotherapy	0.25	0.423 (0.097–1.84)
Solitary metastasis	0.21	0.534 (0.206–1.42)
Small cumulative PIV (≤5 mL)	0.15	0.438 (0.144–1.33)
Two-session SRS	0.26	0.426 (0.095–1.91)

*SRS* stereotactic radiosurgery, *CI* confidence interval, *KPS* Karnofsky performance scale, *PIV* prescription isodose volume

**Fig. 3:**
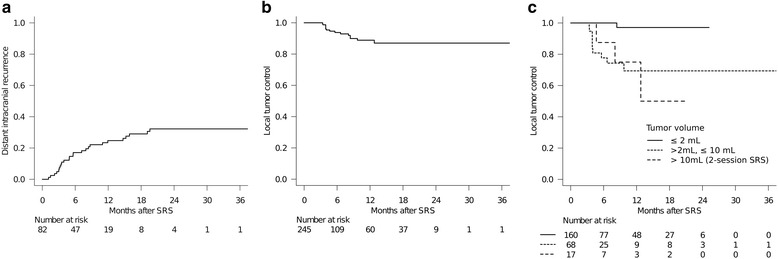
Distant intracranial recurrence rate (**a**), overall local tumor control rate (**b**) and local tumor control rates stratified according to the tumor volume (TV) (**c**). Six-month and 12-month distant intracranial recurrence rates (per patient) were 17% and 25%, respectively. Six-month and 12-month local tumor control rates (per lesion) were 94% and 89%, respectively. TV larger than 2 mL was the only factor predicting a higher rate of local control failure (HR: 12.8, *P* = 0.003)

Only the 245/311 tumors (79%) in 82 patients (77%) who had sufficient radiological follow-up data were analyzed herein because the other 24 patients died from systemic disease progression without follow-up MR imaging studies. Distant metachronous BM were observed in 25 patients (24%). Six-month and 12-month distant BM recurrence rates (per patient) after SRS were 17% and 25%, respectively (Fig. 3a). Six-month and 12-month local tumor control rates (per lesion) were 94% and 89%, respectively (Fig. 3b). Fifteen BM were eventually diagnosed as local tumor recurrence at a median time of 4.7 months after SRS (range: 3.4–12.8 months). A sub-analysis of 2-session SRS conducted for large tumors found a durable TV reduction coupled with symptom relief for 14 of 17 tumors (82%). Three large tumors recurred after initially being responsive to SRS and salvage SRS was thus conducted between 4.7 months and 12.8 months after the initial treatment. The proportional hazards model demonstrated TV larger than 2 mL (HR: 12.8, 95% CI: 2.32–69.3, *P* = 0.003) to be the only factor predicting a higher rate of local control failure (Table 5) (Fig. 3c).

**Table 5:** Analysis of factors predicting local tumor control failure (Cox proportional hazards model)

Covariate	*P* value	Hazard ratio (95% CI)
Large TV (>2 mL)	0.003	12.8 (2.36–69.3)
Tumor causing focal deficit	0.379	1.69 (0.526–5.41)
High marginal dose (≥20Gy)	0.949	1.04 (0.317–3.41)
Two-session SRS	0.946	1.05 (0.284–3.86)

*CI* confidence interval, *TV* tumor volume, *SRS* stereotactic radiosurgery

Twenty-five patients (24%) required repeat SRS for distant or local BM recurrence. The total number of SRS sessions ranged up to 8 and the total number of BM treated per patient ranged up to 42. Four patients (4%) underwent salvage WBRT between 2.6 and 22.6 months after SRS because of subsequent development of miliary BM and/or leptomeningeal dissemination. One patient had to undergo surgical resection under a provisional diagnosis of symptomatic local recurrence 6.6 months after SRS and the histopathological diagnosis was radiation necrosis which predominated over viable adenocarcinoma.

Regarding adverse radiation effects, we experienced one case of radiation-induced optic nerve neuropathy (CTCAE grade 4), secondary to salvage SRS for metachronous recurrence around the left optic canal. Prior to intervention, this patient had been informed of the predicted risk to the affected optic nerve and consented to undergo the intervention. Repeat seizures occurred and newly required anticonvulsive therapy in 3 patients (CTCAE grade 3). Two patients required prolonged oral steroids for delayed symptomatic radiation necrosis (CTCAE grade 2). Tumor-related hemorrhage was observed in 5 patients (3 melanomas, 1 lung adenocarcinoma and 1 renal cell carcinoma) and one of these was mildly symptomatic but eventually showed clinical and radiological stabilization (CTCAE grade 2).

## Discussion

### Current demographic changes as the rationale for the present study

Over the past few decades, the longest extension in life expectancy worldwide has been observed in Japan [24]. World Health Statistics 2014 published by the WHO showed the life expectancies at birth of Japanese men and women to both exceed 80 years [25]. The definition of elderly, when discussing patients with cancer, varies. In many previous studies of SRS for elderly patients with BM, the cut-off ages were set between 65 and 75 years (Table 1) [12–14, 16]. In our country, cancer patients in their 70s are no longer seen as the elderly requiring special care and significant proportions of those age 80 years and older still receive active anti-cancer treatments, as described in the present study. Together with advances in the development of systemic treatments, the long-term control of intracranial disease has become increasingly important not only for overall disease control but also for the patient’s quality of life. However, it is not uncommon for elderly patients to have multiple, concurrent diseases restricting their physiological reserves as well as age-related cognitive decline [16]. Elderly patients with cancer are, in general, less likely to receive definitive therapy and their decisions about treatment may also be influenced by nonmedical, potentially correctable factors such as impaired social services support for those receiving treatment [26].

Few, if any, studies have investigated SRS treatment results for very elderly patients, i.e. those at least 80 years of age, with BM [15] and this aged population has been under-represented in clinical trials of cancer therapy, even in the era of targeted therapy. Thus, the authors considered the age of 80 years to be a reasonable cut-off point, given that the present study aimed to investigate the efficacy and the limitations of comprehensive management of SRS for BM in the very elderly patient population.

### Survival after SRS of elderly patients with BM

The MST slightly longer than 7 months demonstrated herein appears to be shorter than in previous studies of SRS series investigating different patient cohorts conducted in the authors’ institutions [23, 27, 28]. This observation may support age being an important prognostic factor in the majority of patients with malignant primary or metastatic brain tumors, [2, 3]. Watanabe et al. also demonstrated in their case-matched study that post-SRS MST was, in fact, slightly shorter in patients 80 years of age and older than in those 65–79 years of age, although the difference did not reach statistical significance. The OS results after SRS in the present study seemed to be comparable to those of previous studies (Table 1). Minniti et al. reported an exceptionally better OS rate than in other studies. This might be attributable to patient selection criteria such as their adoption of an age cut-off of 70 years, oligometastases (1–4 BM), exclusion of small cell lung cancer, and so on. The present study placed priority on generalizability by including all consecutive cases, even those with large multiple BM and/or very low KPS, and thus reflects the contemporary situation of patients with BM in the community.

### Prognostic factors and selection of candidates for SRS among elderly patients with BM

Identifying factors predicting longer survival in patients with BM is critically important for assigning patients to the optimal treatment modality. In our patient cohort, higher KPS (≥70) (Fig. 2a), controlled primary disease/no extracranial metastases (Fig. 2b) and female sex (Fig. 2c) were independently associated with better patient survival in multivariate analyses (Table 3). High KPS scores and systemic disease control have already been validated in large prospective datasets from radiation therapy oncology group (RTOG) trials [2, 5], as well as being reproduced in prior studies focusing on elderly patients (Table 1). Regarding female sex, we speculate that this might be attributable to the difference in the prevalence of molecular targeted therapy use between males and females. In our patient cohort, 14 of 16 patients receiving molecular targeted therapy were female. Although not shown in the results because these observations are quite preliminary, patients treated with molecular targeted therapy had longer survival than those not receiving such treatments (16.9 months vs. 5.8 months, log-rank test, *P* = 0.007). The emerging role of combining SRS and molecular targeted therapy merits future investigation. Our observations suggest that selected subsets of patients can be expected to experience prolonged survival, although the expected survival may be limited in the majority of elderly patients with BM. The long interval from primary diagnosis to SRS was of borderline significance in the multivariate analysis (*P* = 0.053). This might, at least in part, be attributable to patients with a long prior disease history having been self-selected to do well by virtue of having had time to develop BM and not dying of their systemic disease due to inherently indolent cancers.

### The mode of death and its clinical significance

Concerning the cause of death, many of these patients actually died of extracranial disease progression, as demonstrated herein. Given this observation, OS may not be an appropriate endpoint for accurately evaluating the efficacy and limitations of SRS for BM. The authors believe it to be important to measure how SRS might delay or even prevent worsening neurological symptoms and eventually neurological death regardless of the patient’s age, while adequately maintaining the patient’s quality of life. From this viewpoint, clinical information about the mode of death and the local control of BM is indispensable. Understanding potential differences in the mode of death, is anticipated to facilitate answering the important question of whether treating BM delays neurological progression long enough to allow for a comfortable remaining life. The present study showed that neurological death could be delayed or even prevented by SRS in the majority of very elderly patients with BM, although the observed OS was still limited. Unfortunately, no risk factors clearly associated with neurological death were identified herein (Table 3), probably due to the lack of events of interest. Further experience needs to be accumulated to identify factors potentially influencing neurological death.

### Importance of follow-up management and salvage treatment strategies

The local tumor control rate demonstrated herein appeared to be acceptable and was similar to those obtained in previous studies (Table 1) [12–15]. Regarding factors influencing local tumor control in the present study, mid-size to large tumors (>2 mL) were more likely to recur or complicate radiation-induced toxic events (Table 4). This finding supports prior SRS studies showing TV to be an important predictor of local control in patients with BM treated with single-dose SRS [22, 29, 30]. The present study failed to demonstrate the relationship between prescription dose and local control rate. We speculate that one of the reasons might involve the validity of dose estimation for 2-session SRS based on the LQ model, which is applied to adjust for the difference between a single session and two sessions. There has been controversy as to whether the LQ model is appropriate for large doses per fraction. Brown et al. recently reported that, for most tumors, the LQ model is still relevant for explaining the results obtained from clinical studies of SRS and stereotactic radiotherapy [31]. On the other hand, the possibility of additional biological effects resulting from endothelial cell damage, enhanced tumor immunity, or both has been suggested [32, 33]. However, we do not yet have an appropriate model taking into account these additional factors.

Two-session SRS conducted for large tumors, in fact, achieved an acceptable local control rate (75% at 1 year) although it was lower than that of small metastases (Fig. 3c). Considering the low alpha/beta ratio of the tissue in the central nervous system and the author’s as yet limited experience, hypofractionated stereotactic radiotherapy might be among the potential alternatives for reducing acute toxicities while improving the local control rate in patients with mid-size to large tumors. However, the clinical evidence accumulated to date is not yet conclusive [34, 35]. The potential for improvement of local tumor control using hypofractionated stereotactic radiotherapy with a Gamma Knife unit warrants further research.

Subsequent intervention was actually needed in 26 patients (25%), mostly because of distant BM recurrence. Most of these cases were successfully managed with repeat SRS. Hanssens et al. reported that SRS alone based on high-resolution MR imaging, decreased the incidence of and increased the time until distant recurrences [36]. In fact, the competing risks regression analysis employed herein indicated the rate of salvage treatment for new BM to be somewhat lower than those in prior studies (25% at 1 year) [12–14]. Although there is no general consensus regarding the risks and benefits of omitting upfront WBRT, it appears that repeat radiosurgery may be effective as salvage therapy for recurrent tumors after SRS alone, especially in elderly patients. Taking into account the detrimental delayed effects of WBRT on cognitive function and health-related quality of life [8, 9, 37], it may be a rational treatment approach to strategically withhold WBRT until it would presumably be the most efficient treatment option [9]. To assure the relevance of SRS management, meticulous clinical and neuroimaging follow-up and timely salvage SRS are essential, while it should be noted that such a treatment strategy does have the potential to place a major socio-economic burden on elderly cancer patients and their relatives.

### Weaknesses of the present study

This study has several limitations. The critical issue in the present study is patient selection bias inherent to the retrospective approach. It is possible that elderly patients with limited numbers of BM in the present study had developed tolerance to the treatment and also had better access to our institution and were consequently self-selected to do well. It must be also appreciated that we cannot address the potential role of SRS in comparison to WBRT, given the exploratory nature of the analyses in a non-comparative study. We could not control for the possibility that there may have been patients not sent to us, due to referral bias, whose outcomes could have differed from what was observed in the present patient cohort. In follow-up management, some patients continued to be followed by their referring oncologists. Neuroimaging protocols could have differed among these hospitals, and we cannot rule out the possibility of patients not being referred to our institution even if tiny intracranial local or distant recurrences were detected in those in poor condition with very short life expectancies. Therefore, it is necessary to fully recognize that the rates of local and distant recurrences of BM might be underestimates. In addition, the relatively small number of patients and relative heterogeneity of the patient population may have limited the statistical power of the analyses, leading to incorrect conclusions. More evidence-based information obtained from a well-designed prospective comparative study is needed to confirm our findings regarding the clinical efficacy of SRS for elderly patients with BM.

## Conclusions

We investigated the efficacy of SRS for BM in a cohort of very elderly patients and our findings suggested SRS to be a feasible and effective treatment option even for those of advanced age with BM. Close follow-up and continuation of radiosurgical management might contribute to reducing the rate of neurological death. Prognostic factors associated with better OS in our cohort were high KPS, controlled primary disease/no extracranial metastases and female sex.

## Abbreviations

BM: brain metastases
CI: confidence interval
HR: hazard ratio
KPS: Karnofsky performance status
LQ: linear-quadratic
MR: magnetic resonance
MST: median survival time
NCI-CTCAE: national cancer institute common terminology criteria for adverse events
OS: overall survival
PIV: prescription isodose volume
RPA: recursive partitioning analysis
RTOG: radiation treatment oncology group
SRS: stereotactic radiosurgery
TV: tumor volume
WBRT: whole brain radiotherapy
